# Supplementary material for: Cathepsin D deficiency in mammary epithelium transiently stalls breast cancer by interference with mTORC1 signaling
Source: Nat Commun. 2020 Oct 12;11:5133. doi: 10.1038/s41467-020-18935-2 (PMC7552405; doi:10.1038/s41467-020-18935-2)
Supplement: Supplementary file 1 — Supplementary Information [file 41467_2020_18935_MOESM1_ESM.pdf]

Cathepsin D deficiency in mammary epithelium transiently stalls breast cancer by interference with mTORC1 signaling

Ketterer et al.

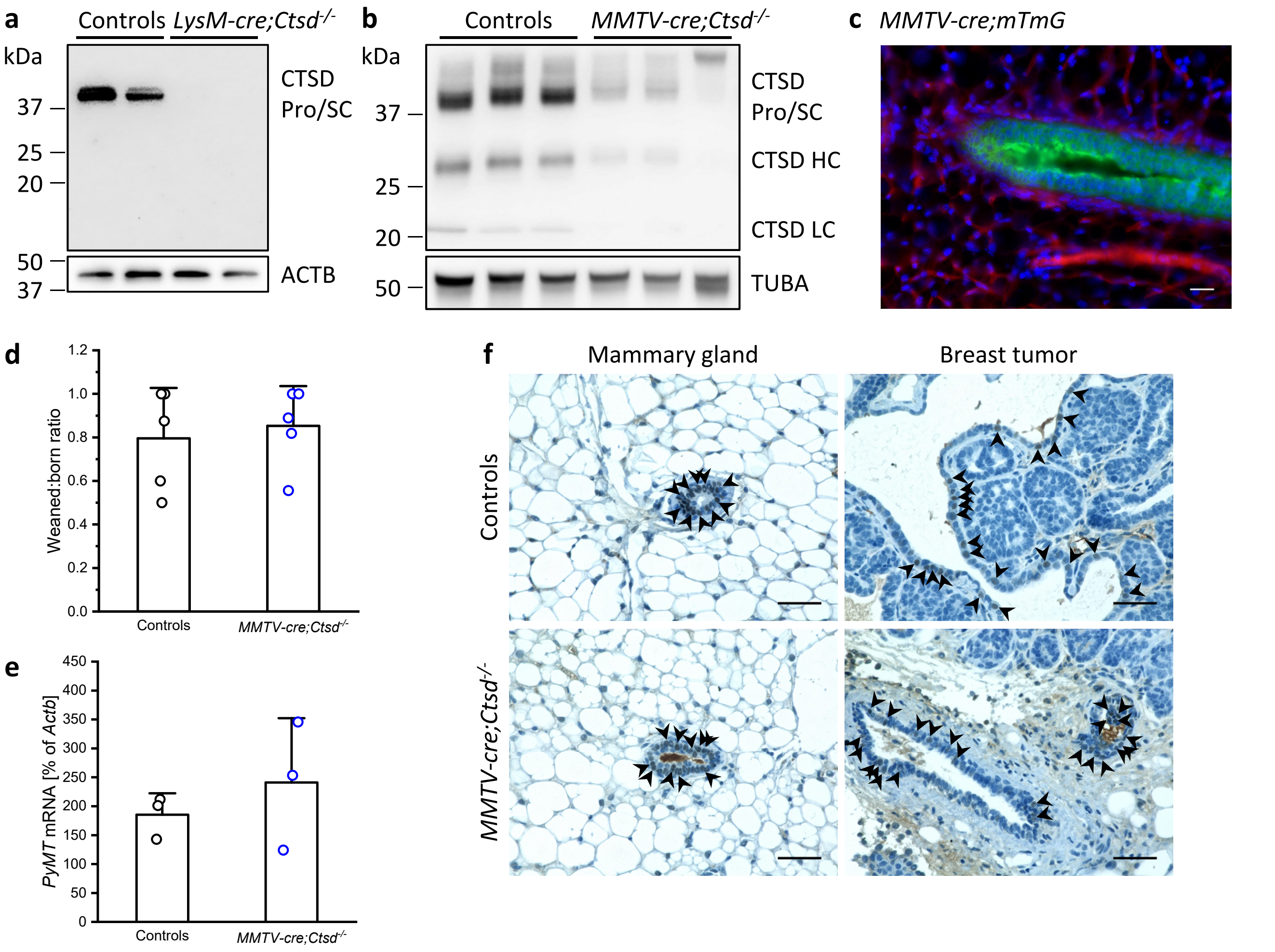

Supplementary Fig. 1 | Myeloid- and mammary epithelium-specific CTSD deficiency. **a**, Analysis of CTSD expression by Western blot (with ACTB as loading control) in bone marrow-derived macrophages from control (one *Ctsd*$^{+/fl}$ + one *Ctsd*$^{fl/fl}$) and *LysM-cre;Ctsd*$^{-/-}$ mice. Pro/SC, zymogen/single-chain form. Two independent experiments. **b**, Analysis of CTSD expression by Western blot (with TUBA as loading control) in end-stage tumors from control (*Ctsd*$^{+/fl}$) and *MMTV-cre;Ctsd*$^{-/-}$ PyMT mice. Pro/SC, zymogen/single-chain form; HC, heavy chain; LC, light chain. Three independent experiments. **c**, Picture of mammary gland section from 7-week-old *MMTV-cre;mTmG* mouse counterstained for DNA (blue). Bar, 50 µm. Representative of 2 independent experiments. **d**, Weaned-to-born ratio of offspring from control (2x *Ctsd*$^{+/fl}$ + 3x *Ctsd*$^{fl/fl}$) and *MMTV-cre;Ctsd*$^{-/-}$ female mice (n = 5 animals). **e**, Relative *PyMT* expression determined by RT-PCR in end-stage tumors from control (*Ctsd*$^{+/fl}$) and *MMTV-cre;Ctsd*$^{-/-}$ PyMT mice (n = 3 animals). **f**, Pictures of mammary gland sections (left) from 12-week-old control (*Ctsd*$^{+/+}$) and *MMTV-cre;Ctsd*$^{-/-}$ mice and end-stage tumor sections (right) from control (*Ctsd*$^{+/fl}$) and *MMTV-cre;Ctsd*$^{-/-}$ PyMT mice stained for estrogen receptor alpha (ERα) by immunohistochemistry. Representative ERα-positive cells are highlighted by arrowheads. Bars, 50 µm. Representative of 2 independent experiments. Bar charts show all data points with mean + SD. Source data are provided as a Source Data file.

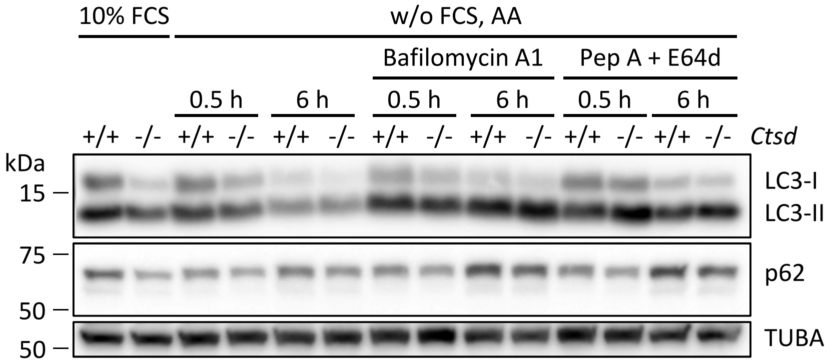

Supplementary Fig. 2 | CTSD deficiency increases the autophagic flux in short-term starved tumor cells. Analysis of LC3-I, LC3-II, and p62 protein levels by Western blot (with TUBA as loading control) in *Ctsd*$^{+/+}$ and *Ctsd*$^{-/-}$ PyMT cells cultured for 0.5 or 6 h in 10% FCS or FCS- and amino acid (AA)-free medium supplemented with the vATPase inhibitor Bafilomycin A1 or a combination of the aspartic protease inhibitor Pepstatin A (Pep A) and the cysteine protease inhibitor E64d. Representative of 3 independent experiments. Source data are provided as a Source Data file.

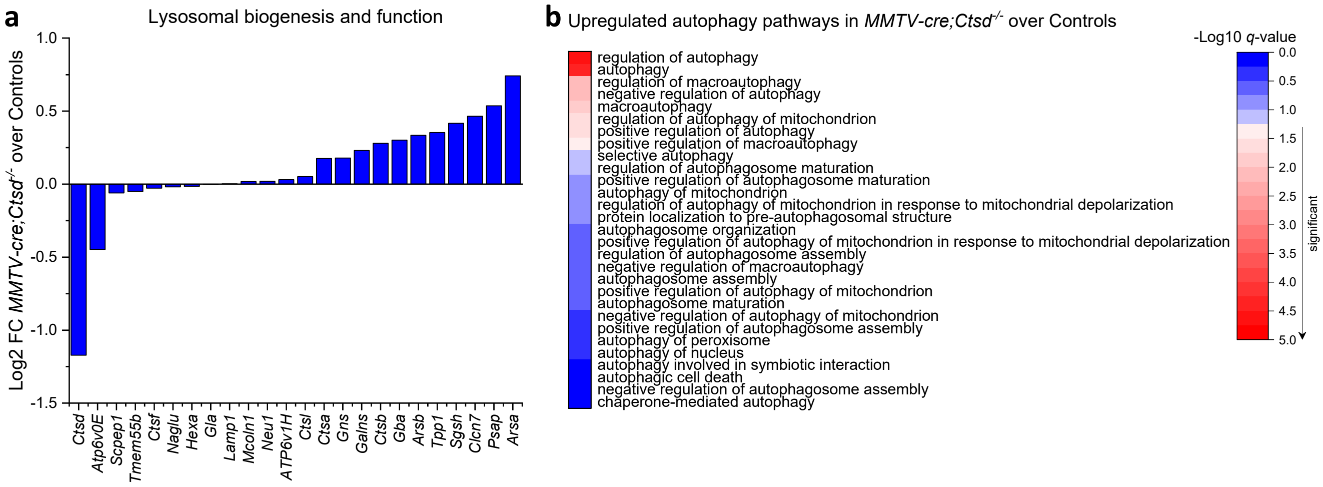

Supplementary Fig. 3 | Increased autolysosomal compartment in tumors from *MMTV-cre;Ctsd$^{-/-}$* PyMT mice. a, Change in expression of genes of the lysosomal biogenesis and function gene set comparing the transcriptome of tumors from 18-week-old *MMTV-cre;Ctsd$^{-/-}$* and control (*Ctsd$^{fl/fl}$*) PyMT mice, plotted as log2 fold change (FC) (*n* = 4 animals). **b**, Gene Ontology (GO)-based Gene Set Enrichment Analysis (GSEA) of differentially expressed genes in *MMTV-cre;Ctsd$^{-/-}$* tumors compared to control (*Ctsd$^{fl/fl}$*) tumors with transcriptome data (*n* = 4 animals). *q*-values for upregulated autophagy pathways are shown, *q* < 0.05 labelled as significant. Source data are provided as a Source Data file.

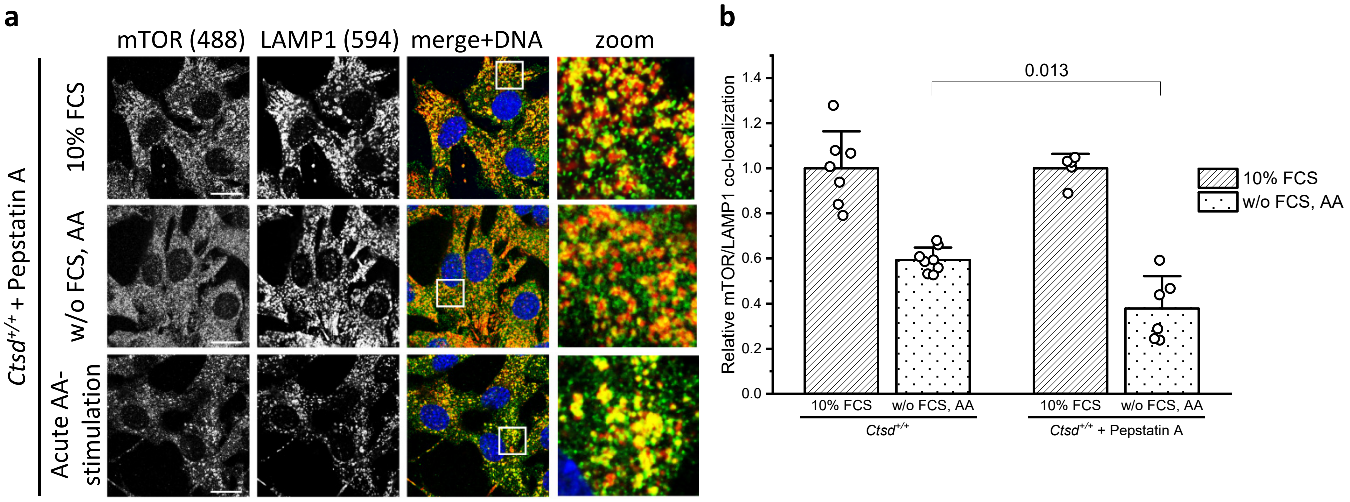

Supplementary Fig. 4 | Pepstatin A treatment of CTSD-competent tumor cells. a, Pictures of 10% FCS, FCS- and amino acid (AA)-starved, and acutely AA-stimulated *Ctsd*$^{+/+}$ PyMT cells treated with the aspartic protease inhibitor Pepstatin A and stained for mTOR (green), LAMP1 (red), and DNA (blue). Zoom shows enlargement of indicated image area in merge+DNA. Bars, 10 µm. Representative of 2 independent experiments. **b**, Quantification of mTOR/LAMP1 co-localization in Pepstatin A-treated *Ctsd*$^{+/+}$ PyMT cells. Pearson correlation coefficient of FCS- and AA-starved cells relative to 10% FCS cells (*n* = 5 images for Pepstatin A-treated *Ctsd*$^{+/+}$ in 10% FCS, *n* = 6 images for Pepstatin A-treated *Ctsd*$^{+/+}$ w/o FCS, AA; two independent experiments; two-sided two-sample t-test). The analysis of untreated *Ctsd*$^{+/+}$ PyMT cells, presented in Fig. 6g, is included for convenient comparison. Bar chart shows all data points with mean + SD and *p*-value. Source data are provided as a Source Data file.

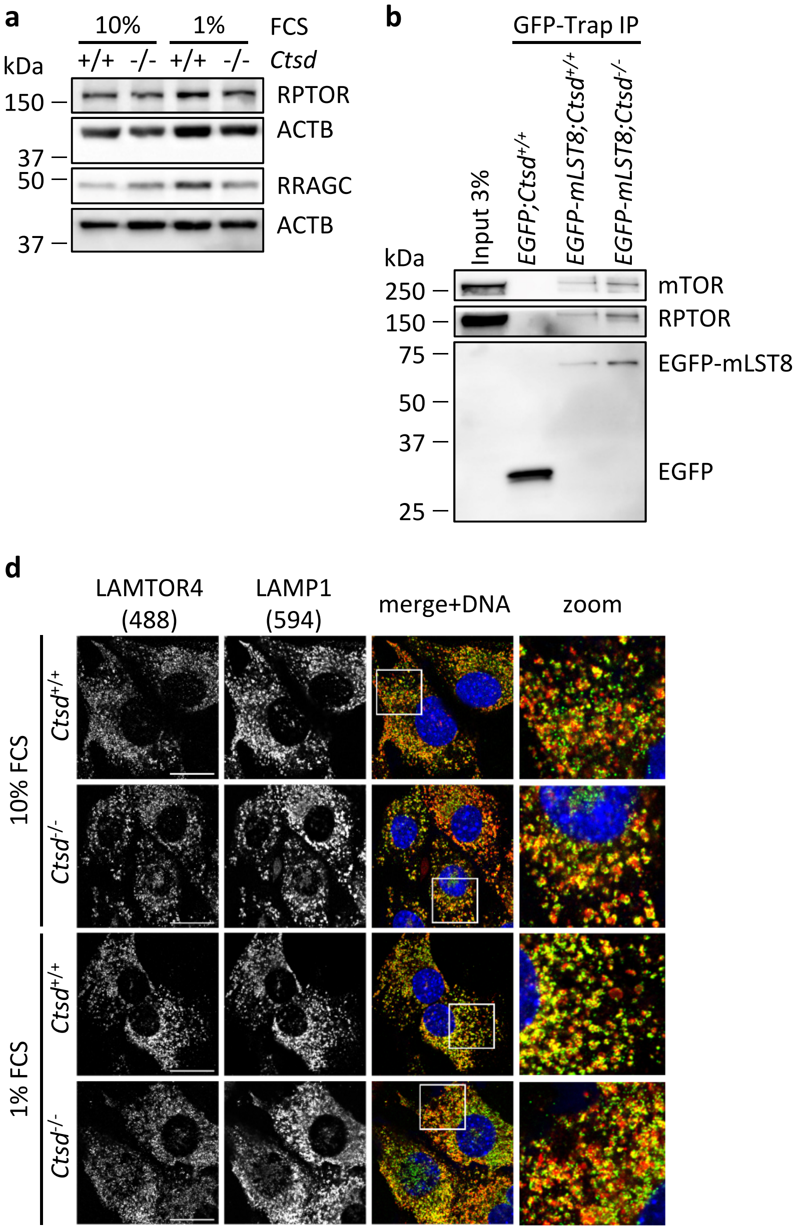

Supplementary Fig. 5 | mTORC1 assembly and lysosome-associated interaction partners. a, Analysis of RPTOR and RRAGC expression by Western blot (with ACTB as loading control) in *Ctsd*$^{+/+}$ and *Ctsd*$^{-/-}$ PyMT cells cultured for 7-10 days in 10% FCS or 1% FCS medium. Representative of 2 (RPTOR) and 3 (RRAGC) independent experiments. **b**, Analysis of mTOR and RPTOR immunoreactivity by Western blot (with EGFP as loading control) in GFP immunoprecipitates (IP) from *Ctsd*$^{+/+}$ and *Ctsd*$^{-/-}$ PyMT cells transduced with EGFP or EGFP-mLST8, respectively. Representative of 2 independent experiments. **c**, Analysis of RHEB and LAMTOR4 expression by Western blot (with TUBA and ACTB as loading control) in 10% FCS and 1% FCS *Ctsd*$^{+/+}$ and *Ctsd*$^{-/-}$ PyMT cells. Representative of 2 (LAMTOR4) and 3 (RHEB) independent experiments. **d**, Pictures of 10% FCS and 1% FCS *Ctsd*$^{+/+}$ and *Ctsd*$^{-/-}$ PyMT cells stained for LAMTOR4 (green), LAMP1 (red), and DNA (blue). Zoom shows enlargement of indicated image area in merge+DNA. Bars, 10 µm. Representative of one experiment. Source data are provided as a Source Data file.

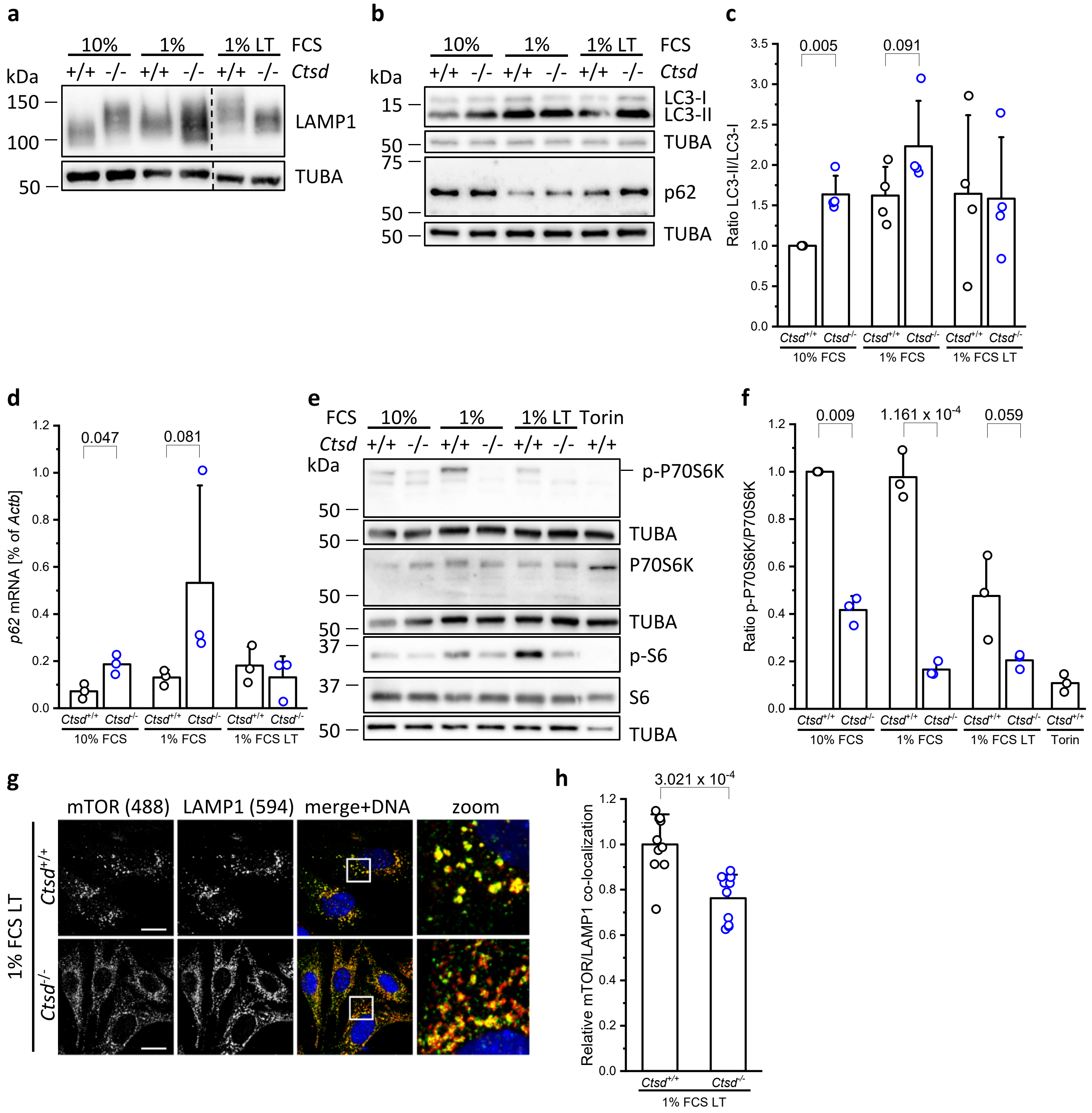

Supplementary Fig. 6 | Autolysosomal compartment and mTORC1 signaling in 1% FCS LT tumor cells. Analysis of LAMP1 (**a**), LC3-I, LC3-II, and p62 (**b**) protein levels by Western blot (with TUBA as loading control) in *Ctsd*$^{+/+}$ and *Ctsd*$^{-/-}$ PyMT cells cultured for ≥ 8 weeks in 1% FCS medium (1% FCS LT). Representative of 2 (LAMP1) and 4 (LC3, p62) independent experiments. **c**, Quantification of LC3 Western blots as in (b), plotted as LC3-II/LC3-I ratio relative to 10% FCS *Ctsd*$^{+/+}$ (n = 4 independent experiments; two-sided one-sample and two-sample t-test). **d**, Relative *p62* expression determined by RT-PCR in 1% FCS LT *Ctsd*$^{+/+}$ and *Ctsd*$^{-/-}$ PyMT cells (n = 3 independent experiments; two-sided two-sample t-test). **e**, Analysis of phosphorylated (p-P70S6K and p-S6) and total P70S6K and S6 protein levels by Western blot (with TUBA as loading control) in 1% FCS LT *Ctsd*$^{+/+}$ and *Ctsd*$^{-/-}$ PyMT cells. Representative of 2 ((p-)S6) and 3 ((p-)P70S6K) independent experiments. **f**, Quantification of (p-)P70S6K Western blots as in (e), plotted as p-P70S6K/P70S6K ratio relative to 10% FCS *Ctsd*$^{+/+}$ (n = 3 independent experiments; two-sided one-sample and two-sample t-test). **g**, Pictures of 1% FCS LT *Ctsd*$^{+/+}$ and *Ctsd*$^{-/-}$ PyMT cells stained for mTOR (green), LAMP1 (red), and DNA (blue). Zoom shows enlargement of indicated image area in merge+DNA. Bars, 10 µm. Representative of 2 independent experiments. **h**, Quantification of mTOR/LAMP1 co-localization in (g) (n = 10 images; two independent experiments; two-sided two-sample t-test). Short-term conditions (10% FCS, 1% FCS, Torin) as presented in Fig. 4b, Fig. 5a, b, d and Fig. 6b, c are included for convenient comparison. Bar charts show all data points with mean + SD and p-value. Source data are provided as a Source Data file.

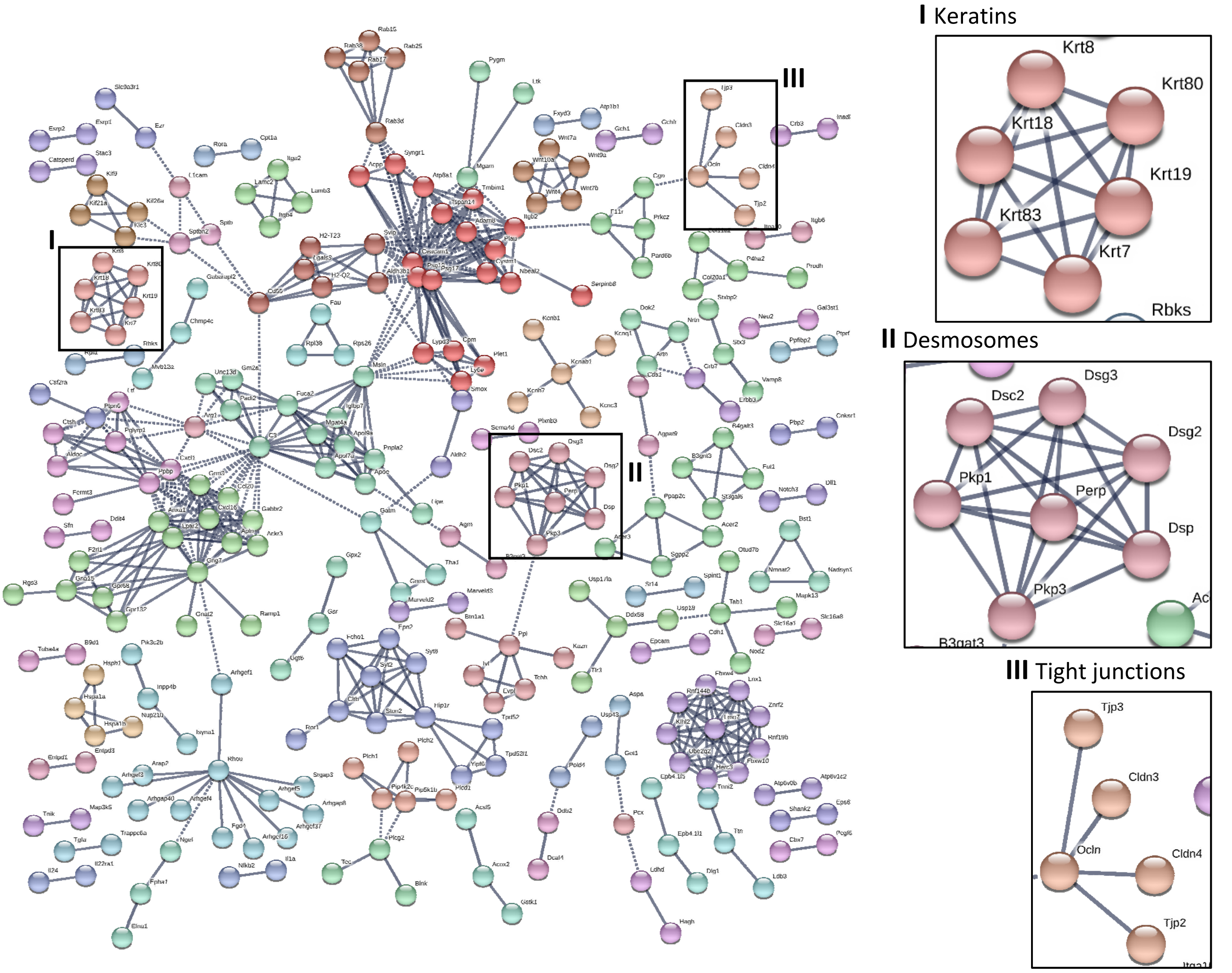

Supplementary Fig. 7 | Re-growing CTSD-deficient tumor cells downregulate epithelial genes. Search Tool for the Retrieval of Interacting Genes/Proteins (STRING) -based cluster analysis of 1066 transcripts expressed and significantly downregulated in *Ctsd*$^{-/-}$ PyMT cells cultured for ≥ 8 weeks in 1% FCS medium (1% FCS LT) compared to 1% FCS *Ctsd*$^{-/-}$ PyMT cells and corrected for long-term 1% FCS culture effects in *Ctsd*$^{+/+}$ PyMT cells (*n* = 3 independent experiments). Clusters containing epithelial genes (I-III) are magnified. Source data are provided as a Source Data file.

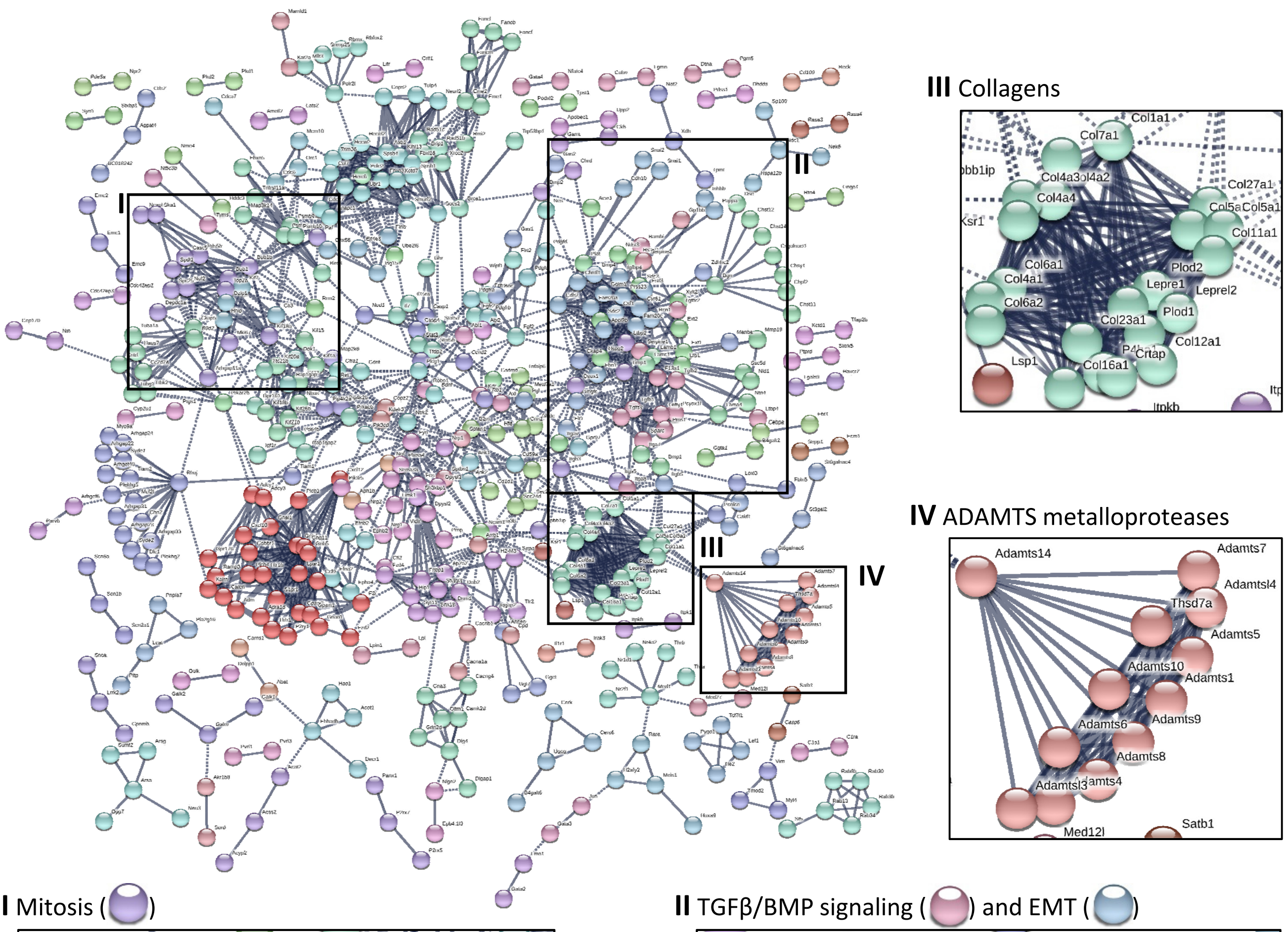

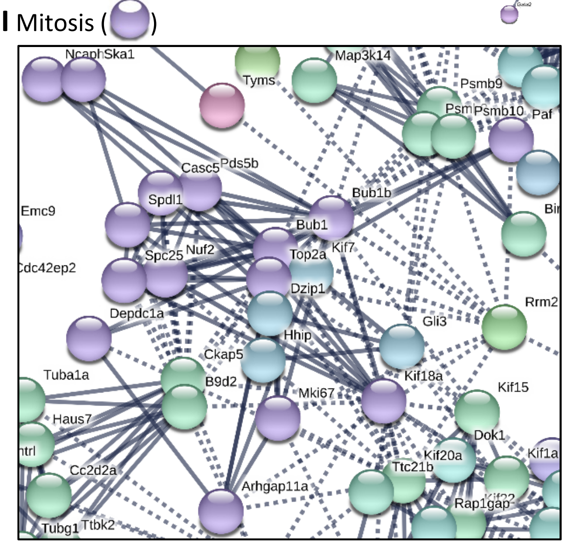

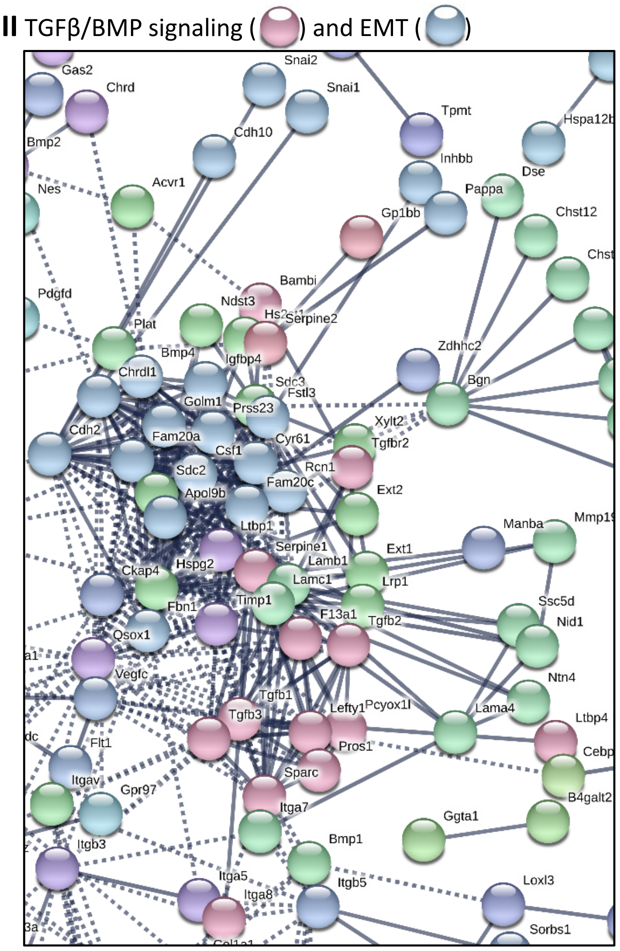

Supplementary Fig. 8 | Re-growing CTSD-deficient tumor cells upregulate mitotic and mesenchymal genes. Search Tool for the Retrieval of Interacting Genes/Proteins (STRING) -based cluster analysis of 1429 transcripts expressed and significantly upregulated in *Ctsd*$^{-/-}$ PyMT cells cultured for ≥ 8 weeks in 1% FCS medium (1% FCS LT) compared to 1% FCS *Ctsd*$^{-/-}$ PyMT cells and corrected for long-term 1% FCS culture effects in *Ctsd*$^{+/+}$ PyMT cells (*n* = 3 independent experiments). Clusters containing mitotic (I) and mesenchymal genes (II-IV) are magnified. Source data are provided as a Source Data file.

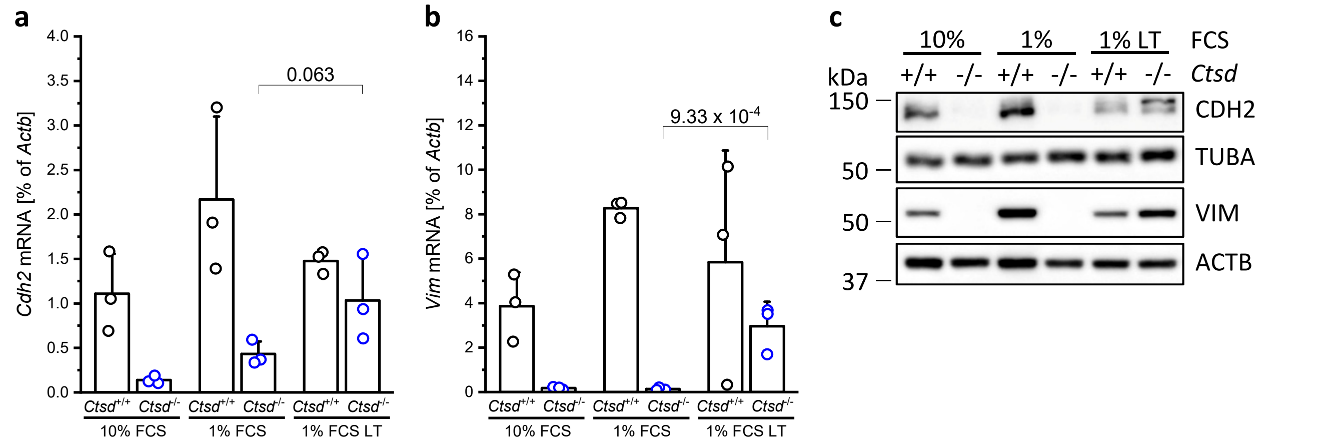

Supplementary Fig. 9 | CTSD-deficient tumor cells induce mesenchymal markers during re-growth. Relative *Cdh2* (**a**) and *Vim* (**b**) expression determined by RT-PCR in $Ctsd^{+/+}$ and $Ctsd^{-/-}$ PyMT cells cultured for 7-10 days in 10% FCS or 1% FCS medium or for ≥ 8 weeks (1% FCS LT) in 1% FCS medium (*n* = 3 independent experiments; two-sided two-sample t-test). **c**, Analysis of CDH2 and VIM protein levels by Western blot (with TUBA and ACTB as loading control) in 10% FCS, 1% FCS, and 1% FCS LT $Ctsd^{+/+}$ and $Ctsd^{-/-}$ PyMT cells. Representative of 2 independent experiments. Bar charts show all data points with mean + SD and *p*-value. Source data are provided as a Source Data file.

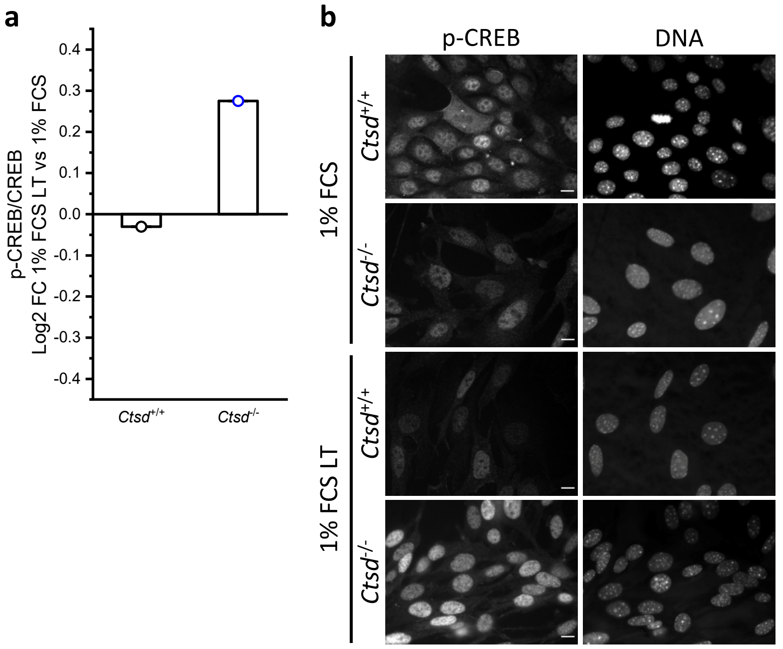

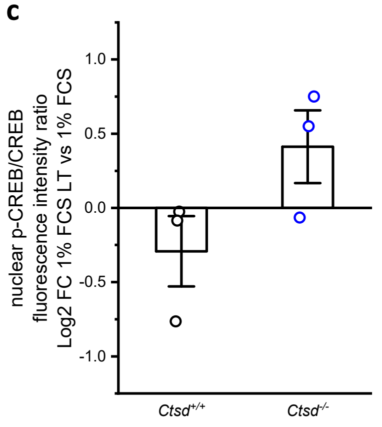

d

Effective concentration of KG-501 [µM]		
	Ctsd $^{+/+}$	***Ctsd*** $^{-/-}$
EC_{20}	13.1	10.6
EC_{50}	6.2	5.0
EC_{80}	2.9	2.4

Supplementary Fig. 10 | Increased phosphorylation of CREB in re-growing CTSD-deficient tumor cells. a, Quantification of phosphorylated (p-CREB) and total CREB protein levels by alphaLISA in lysates from *Ctsd* $^{+/+}$ and *Ctsd* $^{-/-}$ PyMT cells cultured for 7-10 days (1% FCS) or for ≥ 8 weeks (1% FCS LT) in 1% FCS medium. The p-CREB/CREB ratio is plotted as log2 fold change (FC) of 1% FCS LT versus 1% FCS (*n* = 1 experiment). **b**, Pictures of 1% FCS and 1% FCS LT *Ctsd* $^{+/+}$ and *Ctsd* $^{-/-}$ PyMT cells stained for p-CREB and DNA. Bars, 10 µm. Representative of 3 independent experiments. **c**, Quantification of the nuclear fluorescence intensity of p-CREB (shown in b) and CREB, whose ratio is plotted as log2 FC of 1% FCS LT versus 1% FCS for *Ctsd* $^{+/+}$ and *Ctsd* $^{-/-}$ PyMT cells (*n* = 3 independent experiments). **d**, Effective concentrations of the CREB inhibitor KG-501 for 1% FCS LT *Ctsd* $^{+/+}$ and *Ctsd* $^{-/-}$ PyMT cells determined by MTT assay (*n* = 2 independent experiments). Bar charts show all data points with mean ± SEM. Source data are provided as a Source Data file.

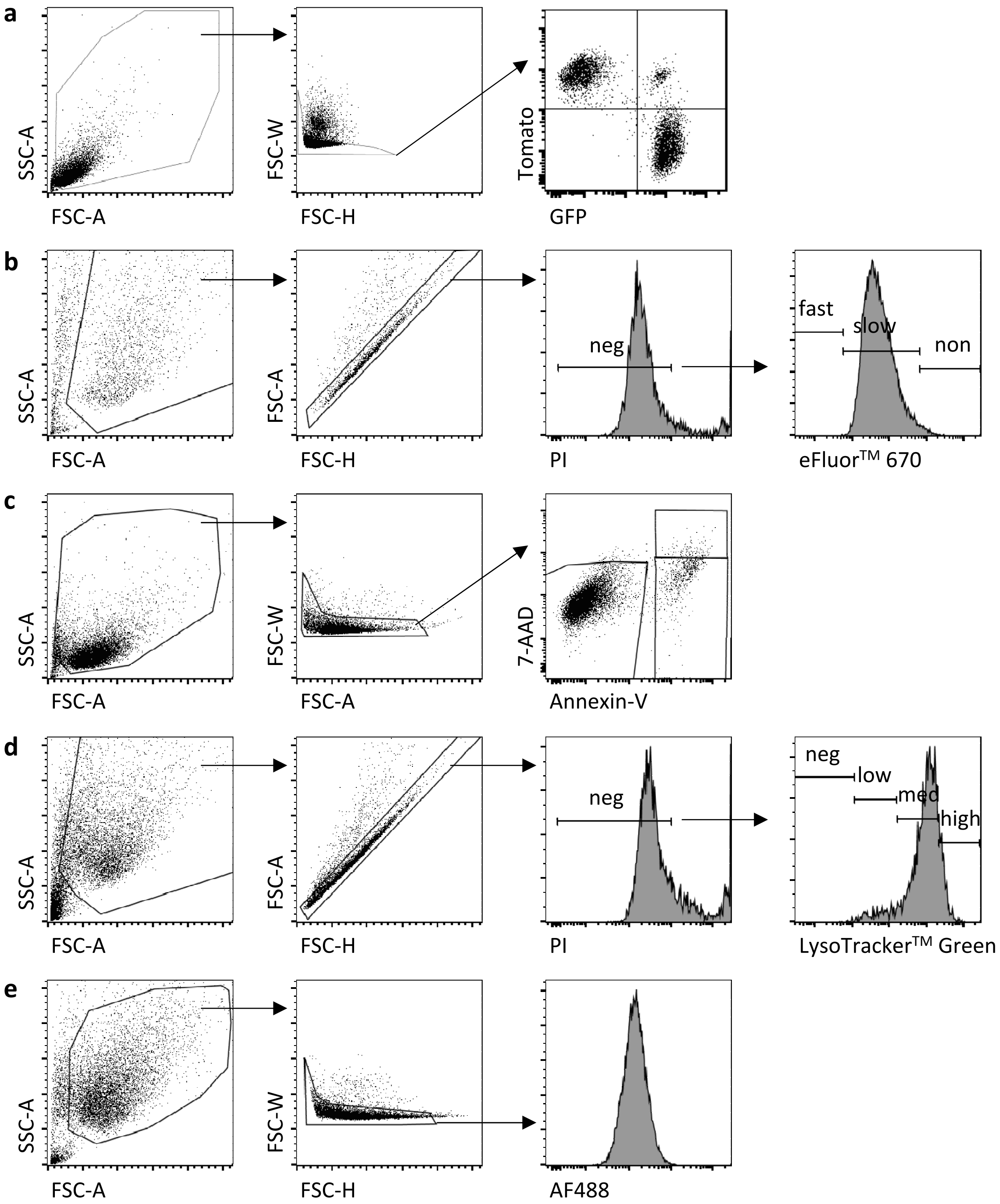

Supplementary Fig. 11 | Flow cytometry of tumor cells. Gating strategy to determine (**a**) the percentage of non-recombined (Tomato$^+$) and recombined (Tomato$^+$GFP$^+$ and GFP$^+$) cells in competitive growth assays presented in Fig. 2a, c-e, (**b**) the percentage of PI$^-$ fast, slow and non-proliferating cells in label-retention assays presented in Fig. 3c and Fig. 7f, (**c**) the percentage of living (Annexin-V$^-$7-AAD$^-$) cells in apoptosis assays presented in Fig. 3d, (**d**) the percentage of PI$^-$ cells with negative, low, medium and high LysoTracker™ Green intensity for the assessment of acidic cell compartments presented in Fig. 4a and (**e**) the median fluorescence intensity of cells incubated with a self-quenched substrate in lysosomal activity assays presented in Fig. 4f.
