# Supplementary material for: Cathepsin D deficiency in mammary epithelium transiently stalls breast cancer by interference with mTORC1 signaling
Source: Nat Commun. 2020 Oct 12;11:5133. doi: 10.1038/s41467-020-18935-2 (PMC7552405; doi:10.1038/s41467-020-18935-2)
Supplement: Supplementary file 3 — Reporting Summary [file 41467_2020_18935_MOESM3_ESM.pdf]

nature research

Corresponding author(s): Thomas Reinheckel

Last updated by author(s): Sep 15, 2020

Reporting Summary

Nature Research wishes to improve the reproducibility of the work that we publish. This form provides structure for consistency and transparency in reporting. For further information on Nature Research policies, see our Editorial Policies and the Editorial Policy Checklist.

Statistics

For all statistical analyses, confirm that the following items are present in the figure legend, table legend, main text, or Methods section.

n/a	Confirmed	
☐	☒	The exact sample size (*n*) for each experimental group/condition, given as a discrete number and unit of measurement
☐	☒	A statement on whether measurements were taken from distinct samples or whether the same sample was measured repeatedly
☐	☒	The statistical test(s) used AND whether they are one- or two-sided<br>*Only common tests should be described solely by name; describe more complex techniques in the Methods section.*
☒	☐	A description of all covariates tested
☐	☒	A description of any assumptions or corrections, such as tests of normality and adjustment for multiple comparisons
☐	☒	A full description of the statistical parameters including central tendency (e.g. means) or other basic estimates (e.g. regression coefficient) AND variation (e.g. standard deviation) or associated estimates of uncertainty (e.g. confidence intervals)
☐	☒	For null hypothesis testing, the test statistic (e.g. *F*, *t*, *r*) with confidence intervals, effect sizes, degrees of freedom and *P* value noted<br>*Give P values as exact values whenever suitable.*
☒	☐	For Bayesian analysis, information on the choice of priors and Markov chain Monte Carlo settings
☒	☐	For hierarchical and complex designs, identification of the appropriate level for tests and full reporting of outcomes
☐	☒	Estimates of effect sizes (e.g. Cohen's *d*, Pearson's *r*), indicating how they were calculated

Our web collection on statistics for biologists contains articles on many of the points above.

Software and code

Policy information about availability of computer code

Data collection	Vevo LAB software (V3.1.1), Keyence BZ-9000 Viewer, Nikon DS-L3, FACSDiva (V6.1.2), Leica LAS-X software, Bio-Rad CFX Manager (V3.1), EnSpire software (V4.1), FusionCapt Advance software (V16.08)
Data analysis	Microsoft Office Excel, Vevo LAB software (V3.1.1), Keyence BZ-9000 Analyzer, ImageJ (V1.52p), FlowJo (V10.6.2), Volocity software (V6.3), Bio-Rad CFX Manager (V3.1), FusionCapt Advance software (V16.08), HLImage++ software Quick Spots Tool (V25.0.0), Agilent MassHunter Qualitative Analysis (B.07.00), Agilent MassHunter Quantitative Analysis (B.07.00), MetaboAnalyst (V4.0), Trimmomatic (V0.36), Burrows-wheeler aligner (V0.7.15), VarScan2 (V2.4.3), ANNOVAR (2017-07-17), STAR (V2.5.3a), R/Bioconductor package limma (V3.40.6), STRING (V11.0), OriginPro (2018G-2020)

For manuscripts utilizing custom algorithms or software that are central to the research but not yet described in published literature, software must be made available to editors and reviewers. We strongly encourage code deposition in a community repository (e.g. GitHub). See the Nature Research guidelines for submitting code & software for further information.

Data

Policy information about availability of data

All manuscripts must include a data availability statement. This statement should provide the following information, where applicable:

- Accession codes, unique identifiers, or web links for publicly available datasets
- A list of figures that have associated raw data
- A description of any restrictions on data availability

The exome and transcriptome data have been deposited in the BioProject database under the accession code PRJNA663291 (http://www.ncbi.nlm.nih.gov/bioproject/663291) and in the Gene Expression Omnibus (GEO) database under the accession code GSE133328 (https://www.ncbi.nlm.nih.gov/geo/query/acc.cgi?acc=GSE133328), respectively. The CREB gene set and the list of mutated genes in human breast carcinoma referenced during the study are available in public

repositories from the harmonizome (http://amp.pharm.mssm.edu/Harmonizome/gene_set/CREB/MotifMap+Predicted+Transcription+Factor+Targets) and COSMIC (https://cancer.sanger.ac.uk/cosmic/browse/tissue?hn=carcinoma&in=t&sn=breast&ss=all) website, respectively. The source data underlying Figs 1b, c, e, f, 2, 3c-f, 4, 5a-d, f, g, 6a-c, g, 7a-c, f and 8 and Supplementary Figs 1a, b, d, e, 2, 3, 4b, 5a-c, 6a-f, h, 7, 8, 9, 10a, c and d are provided as a Source Data file. All the other data supporting the findings of this study are available within the article and its supplementary information files and from the corresponding author upon reasonable request. A reporting summary for this article is available as a Supplementary Information file.

Field-specific reporting

Please select the one below that is the best fit for your research. If you are not sure, read the appropriate sections before making your selection.

☒ Life sciences ☐ Behavioural & social sciences ☐ Ecological, evolutionary & environmental sciences

For a reference copy of the document with all sections, see nature.com/documents/nr-reporting-summary-flat.pdf

Life sciences study design

All studies must disclose on these points even when the disclosure is negative.

Sample size	For all tumor mouse studies, the planning of animal numbers aimed to detect a 30% difference of tumor size between genotypes at the p=0.05 level (two-sided) with a power of 0.8. Considering a 25% variation within genotypes the standardized effect size is about 1.2 resulting in a required group size of 10-12 animals (N) per genotype. For the remaining cell culture studies, sample size was determined to be adequate based on the magnitude and consistency of measurable differences between groups. Exact sample size is indicated in the figures and their legends.
Data exclusions	No data were excluded (except for failed experiments, in which the expected signal was not observed in controls).
Replication	Since not all tumor mice mice were available at once, the experiment was replicated in 4 independent animal cohorts, which showed reproducible effects and were pooled afterwards for analysis. Cell experiments were independently and successfully replicated as specified in the figures and their legends. Metabolic and qPCR analyses were sucessfully and independently replicated 3 times each.
Randomization	Female tumor mice were kept as litters regardless of their cathepsin genotype. Allocation to the groups depended on genotyping, but was otherwise random. Cell samples were randomly allocated to treatment by random division into multiple cell culture plates/wells, that were subsequently kept under identical conditions.
Blinding	For blinding, mice, tumor and cell samples were consecutively numbered. Thus, during monitoring of tumor progression, for analyzing protein co-localisation by immunoflourescence, for targeted metabolomics as well as for transcriptome and genome analyses the participating researchers were unaware of the underlying genotype and/or experimental condition.

Reporting for specific materials, systems and methods

We require information from authors about some types of materials, experimental systems and methods used in many studies. Here, indicate whether each material, system or method listed is relevant to your study. If you are not sure if a list item applies to your research, read the appropriate section before selecting a response.

Materials & experimental systems

n/a	Involved in the study	
☐	☒	Antibodies
☐	☒	Eukaryotic cell lines
☒	☐	Palaeontology and archaeology
☐	☒	Animals and other organisms
☒	☐	Human research participants
☒	☐	Clinical data
☒	☐	Dual use research of concern

Methods

n/a	Involved in the study	
☒	☐	ChIP-seq
☐	☒	Flow cytometry
☒	☐	MRI-based neuroimaging

Antibodies

Antibodies used	ACTB (MP 691001, Clone C4), CDH2 (Cell Signaling 4061), CREB (Cell Signaling 9197, Clone 48H2), CTSB (R&D BAF965), CTSD (R&D AF1029), CTSL (R&D AF1515), donkey-anti-rabbit-AF488 (Life Technologies A21206), estrogen receptor alpha (Santa Cruz Biotechnology sc-8005, Clone D-12), GFP (Cell Signaling 2555), goat-anti-mouse-peroxidase (Sigma A0168), goat-anti-rabbit-horseradish peroxidase (BioRad 172-1019), goat-anti-rat-AF594 (Life Technologies A11007), Ki67 (Abcam ab15580), LAMP1 (Abcam ab25245, Clone 1D4B), LAMP1 (Cell Signaling 3243, Clone C54H11), LAMTOR4 (Cell Signaling 12284, Clone D6A4V), LC3 (Cell Signaling 2775), mTOR (Cell Signaling 2983, Clone 7C10), p38 MAPK (Cell Signaling 9212), p62 (Cell Signaling 5114), P70S6K (Enzo ADI-KAP-CC035-E), p-CREB (Cell Signaling 9198, Clone 87G3), p-p38 MAPK (Cell Signaling 9211), p-P70S6K (Cell Signaling 9205), p-S6 (Cell Signaling 4857, Clone 91B2), rabbit-anti-goat-peroxidase (Sigma A5420), RHEB (Cell Signaling 13879, Clone E1G1R), RPTOR (Cell Signaling 2280, Clone 24C12), RRAGC (Cell Signaling 5466, Clone D31G9), S6 (Cell Signaling 2317, 54D2), TUBA (Sigma T9026, Clone DM1A), VIM (BD 550513, Clone RV202)

Validation

ACTB (MP 691001, Clone C4): Host: mouse; Reactivity: all six known vertebrate isoactins as well as Dictyostelium discoideum and Physarum polycephalum actins; Suitable for: ELISA, Immunofluorescence, Immunoblots and Immunohistochemistry; Validation stated on supplier's website: https://www.mpbio.com/eu/08691001-mouse-anti-actin-monoclonal-clone-c4-cf
CDH2 (Cell Signaling 4061): Host: rabbit; Reactivity: Human, Mouse, Rat, Monkey; Suitable for: Western Blotting; Validation stated on supplier's website: https://www.cellsignal.com/products/primary-antibodies/n-cadherin-antibody/4061?Ntk=Products&Ntt=4061
CREB (Cell Signaling 9197, Clone 48H2): Host: rabbit; Reactivity: Human, Mouse, Rat, Monkey, D. melanogaster; Suitable for: Western Blotting, Immunoprecipitation, Immunohistochemistry, Immunofluorescence, Flow Cytometry, Chromatin IP, Chromatin IP-seq, CUT&RUN; Validation stated on supplier's website: https://www.cellsignal.com/products/primary-antibodies/creb-48h2-rabbit-mab/9197?Ntk=Products&Ntt=9197
CTSB (R&D BAF965): Host: Goat; Reactivity: Mouse; Suitable for: Western blot, Immunohistochemistry; Validation done in lab (knockout cell line) and on supplier's website: https://www.rndsystems.com/products/mouse-cathepsin-b-biotinylated-antibody_baf965
CTSD (R&D AF1029): Host: Goat; Reactivity: Mouse; Suitable for: Western Blot, Immunohistochemistry, Immunoprecipitation, Immunocytochemistry; Validation done in lab (knockout cell line) and on supplier's website: https://www.rndsystems.com/products/mouse-cathepsin-d-antibody_af1029
CTSL (R&D AF1515): Host: Goat; Reactivity: Mouse, Rat; Suitable for: Western Blot, Simple Western, Immunohistochemistry; Validation done in lab (knockout cell line) and on supplier's website: https://www.rndsystems.com/products/mouse-rat-cathepsin-l-antibody_af1515
donkey-anti-rabbit-AF488 (Life Technologies A21206): Host: Donkey; Reactivity: Rabbit; Suitable for: Flow Cytometry, Immunocytochemistry, Immunofluorescence, Immunohistochemistry; Validation stated on supplier's website: https://www.thermofisher.com/antibody/product/Donkey-anti-Rabbit-IgG-H-L-Highly-Cross-Adsorbed-Secondary-Antibody-Polyclonal/A-21206
estrogen receptor alpha (Santa Cruz Biotechnology sc-8005, Clone D-12): Host: Mouse; Reactivity: Mouse, Rat, Human; Suitable for: Western blot, Immunoprecipitation, Immunofluorescence, Immunohistochemistry, ELISA; Validation stated on supplier's website: https://www.scbt.com/p/estrogen-receptor-alpha-antibody-d-12
GFP (Cell Signaling 2555): Host: Rabbit; Reactivity: all species expected; Suitable for: Western Blotting, Immunohistochemistry; Validation stated on supplier's website: https://www.cellsignal.com/products/primary-antibodies/gfp-antibody/2555?Ntk=Products&Ntt=2555
goat-anti-mouse-peroxidase (Sigma A0168): Host: goat; Reactivity: mouse; Suitable for: direct ELISA, immunocytochemistry, immunohistochemistry, western blot; Validation stated on supplier's website: https://www.sigmaaldrich.com/catalog/product/sigma/a0168?lang=de&region=DE
goat-anti-rabbit-horseradish peroxidase (BioRad 172-1019): Host: goat; Reactivity: rabbit; Suitable for: colorimetric or chemiluminescent detection; Validation stated on supplier's website: http://www.bio-rad.com/webroot/web/pdf/lsr/literature/Bulletin_9384.pdf
goat-anti-rat-AF594 (Life Technologies A11007): Host: goat; Reactivity: rat; Suitable for: Flow Cytometry, Immunocytochemistry, Immunofluorescence; Validation stated on supplier's website: https://www.thermofisher.com/antibody/product/Goat-anti-Rat-IgG-H-L-Cross-Adsorbed-Secondary-Antibody-Polyclonal/A-11007
Ki67 (Abcam ab15580): Host: rabbit; Reactivity: human; Suitable for: Immunohistochemistry, Immunocytochemistry, Immunofluorescence; Validation stated on supplier's website: https://www.abcam.com/ki67-antibody-ab15580.html
LAMP1 (Abcam ab25245, Clone 1D4B): Host: rat; Reactivity: mouse, human; Suitable for: Immunohistochemistry, Flow cytometry; Validation stated on supplier's website: https://www.abcam.com/lamp1-antibody-1d4b-ab25245.html
LAMP1 (Cell Signaling 3243, Clone C54H11): Host: Rabbit; Reactivity: Human, Mouse, Monkey; Suitable for: Western blotting; Validation stated on supplier's website: https://www.cellsignal.com/products/primary-antibodies/lamp1-c54h11-rabbit-mab/3243?Ntk=Products&Ntt=3243
LAMTOR4 (Cell Signaling 12284, Clone D6A4V): Host: Rabbit; Reactivity: Human, Mouse, Rat, Monkey; Suitable for: Western Blotting, Immunoprecipitation, Immunofluorescence (Immunocytochemistry); Validation stated on supplier's website: https://www.cellsignal.com/products/primary-antibodies/lamtor4-c7orf59-d6a4v-rabbit-mab/12284?Ntk=Products&Ntt=12284
LC3 (Cell Signaling 2775): Host: Rabbit; Reactivity: Human, Mouse, Rat; Suitable for: Western Blotting, Immunofluorescence (Immunocytochemistry), Flow Cytometry; Validation stated on supplier's website: https://www.cellsignal.com/products/primary-antibodies/lc3b-antibody/2775?Ntk=Products&Ntt=2775
mTOR (Cell Signaling 2983, Clone 7C10): Host: Rabbit; Reactivity: Human, Mouse, Rat, Monkey; Suitable for: Western Blotting, Immunohistochemistry, Immunofluorescence (Immunocytochemistry), Flow Cytometry; Validation stated on supplier's website: https://www.cellsignal.com/products/primary-antibodies/mtor-7c10-rabbit-mab/2983?Ntk=Products&Ntt=2983
p38 MAPK (Cell Signaling 9212): Host: Rabbit; Reactivity: Human, Mouse, Rat, Monkey, Guinea Pig; Suitable for: Western Blotting, Immunohistochemistry, Immunofluorescence (Immunocytochemistry), Flow Cytometry; Validation stated on supplier's website: https://www.cellsignal.com/products/primary-antibodies/p38-mapk-antibody/9212?Ntk=Products&Ntt=9212
p62 (Cell Signaling 5114): Host: Rabbit; Reactivity: Human, Mouse, Rat, Monkey; Suitable for: Western Blotting; Validation stated on supplier's website: https://www.cellsignal.com/products/primary-antibodies/sqstm1-p62-antibody/5114?Ntk=Products&Ntt=5114
P70S6K (Enzo ADI-KAP-CC035-E): Host: rabbit; Reactivity: human, mouse, rat, cow; Suitable for: western blot, ELISA, immunoprecipitation; Validation stated on supplier's website: https://www.labome.com/product/Enzo-Life-Sciences/ADI-KAP-CC035-E.html
p-CREB (Cell Signaling 9198, Clone 87G3): Host: Rabbit; Reactivity: Human, Mouse, Rat; Suitable for: Western Blotting, Immunohistochemistry, Immunofluorescence, Flow Cytometry, Chromatin IP, Chromatin IP-seq, CUT&RUN; Validation stated on supplier's website: https://www.cellsignal.com/products/primary-antibodies/phospho-creb-ser133-87g3-rabbit-mab/9198?Ntk=Products&Ntt=9198
p-p38 MAPK (Cell Signaling 9211): Host: Rabbit; Reactivity: Human, Mouse, Rat, Monkey, D. melanogaster, Pig, S. cerevisiae; Suitable for: Western Blotting, Immunoprecipitation, Immunofluorescence (Immunocytochemistry); Validation stated on supplier's website: https://www.cellsignal.com/products/primary-antibodies/phospho-p38-mapk-thr180-tyr182-antibody/9211?Ntk=Products&Ntt=9211
p-P70S6K (Cell Signaling 9205): Host: Rabbit; Reactivity: Human, Mouse, Rat, Monkey; Suitable for: Western Blotting; Validation done in lab (see Fig. 6b) and on supplier's website: https://www.cellsignal.de/products/primary-antibodies/phospho-p70-s6-kinase-thr389-antibody/9205?Ntk=Products&Ntt=9205
p-S6 (Cell Signaling 4857, Clone 91B2): Host: Rabbit; Reactivity: Human, Mouse, Rat; Suitable for: Western Blotting, Immunohistochemistry, Immunofluorescence (Immunocytochemistry); Validation done in lab (see Fig. 6b) and on supplier's website: https://www.cellsignal.com/products/primary-antibodies/phospho-s6-ribosomal-protein-ser235-236-91b2-rabbit-mab/4857?

Ntk=Products&Ntt=4857
rabbit-anti-goat-peroxidase (Sigma A5420): Host: rabbit; Reactivity: goat; Suitable for: direct ELISA, dot blot, immunohistochemistry; Validation stated on supplier's website: https://www.sigmaaldrich.com/catalog/product/sigma/a5420?lang=de&region=DE
RHEB (Cell Signaling 13879, Clone E1G1R): Host: Rabbit; Reactivity: Human, Mouse, Rat, Monkey; Suitable for: Western blotting, Immunoprecipitation; Validation stated on supplier's website: https://www.cellsignal.com/products/primary-antibodies/rheb-e1g1r-rabbit-mab/13879?Ntk=Products&Ntt=13879
RPTOR (Cell Signaling 2280, Clone 24C12): Host: Rabbit; Reactivity: Human, Mouse, Rat, Monkey; Suitable for: Western blotting, Immunoprecipitation; Validation stated on supplier's website: https://www.cellsignal.com/products/primary-antibodies/raptor-24c12-rabbit-mab/2280?Ntk=Products&Ntt=2280
RRAGC (Cell Signaling 5466, Clone D31G9): Host: Rabbit; Reactivity: Human, Mouse, Rat, Monkey; Suitable for: Western Blotting, Immunoprecipitation, Immunohistochemistry; Validation stated on supplier's website: https://www.cellsignal.com/products/primary-antibodies/ragc-d31g9-xp-rabbit-mab/5466?Ntk=Products&Ntt=5466
S6 (Cell Signaling 2317, 54D2): Host: Mouse; Reactivity: Human, Mouse, Rat, Monkey, D. melanogaster; Suitable for: Western Blotting, Immunohistochemistry, Immunofluorescence (Immunocytochemistry), Flow Cytometry; Validation stated on supplier's website: https://www.cellsignal.com/products/primary-antibodies/s6-ribosomal-protein-54d2-mouse-mab/2317?Ntk=Products&Ntt=2317
TUBA (Sigma T9026, Clone DM1A): Host: mouse; Reactivity: bovine, rat, yeast, human, mouse, chicken, fungi, amphibian; Suitable for: Immunofluorescence, Western blot; Validation stated on supplier's website: https://www.sigmaaldrich.com/catalog/product/sigma/t9026?lang=de&region=DE
VIM (BD 550513, Clone RV202): Host: Mouse; Reactivity: Human, Rat, Mouse, Hamster, Monkey, Dog, Chicken, Rabbit, Cow, Goat; Suitable for: Western blot, Immunohistochemistry, Immunofluorescence, Electron microscopy; Validation stated on supplier's website: https://www.bdbiosciences.com/us/applications/research/stem-cell-research/cancer-research/human/purified-mouse-anti-vimentin-rv202/p/550513

Eukaryotic cell lines

Policy information about cell lines

Cell line source(s)

Murine breast cancer cell lines were specifically generated for this study. The retrovirus packaging cell lines Plat-E and HEK293 were obtained from Cell Biolabs and ATCC, respectively.

Authentication

Murine breast cancer cell lines were authenticated by PCR assays using species- and gene-specific primers that detect the genetic modifications during generation of the genetically engenieered mice. Plat-E and HEK293 cells were successfully used for retrovirous production and not further authenticated in our lab.

Mycoplasma contamination

Mycoplasma contamination was tested and absent in all cells used in this study.

Commonly misidentified lines (See ICLAC register)

No commonly misidentified cell lines were used in this study.

Animals and other organisms

Policy information about studies involving animals; ARRIVE guidelines recommended for reporting animal research

Laboratory animals

Female mice of the following mouse strains were used at ages of up to 30 weeks: C57BL/6N-Ctsdfl/fl, C57BL/6J-Tg(pCA-mT/mG), B6.129P2-Lyz2tm1(cre)Ifo/J, C57BL/6N-Tg(MMTV-cre), C57BL/6N-Tg(MMTV-PyMT), BALB/c-Rag2-/-;γc-/-. Exact ages and analysis time points are indicated in the article.

Wild animals

No wild animals were used in this study.

Field-collected samples

No field-collected samples were used in this study.

Ethics oversight

All animal procedures were approved by the legal authorities and ethics committee at the regional council Freiburg (registration number: G14/18) and were performed in accordance with the German law for animal welfare.

Note that full information on the approval of the study protocol must also be provided in the manuscript.

Flow Cytometry

Plots

Confirm that:

☒ The axis labels state the marker and fluorochrome used (e.g. CD4-FITC).

☒ The axis scales are clearly visible. Include numbers along axes only for bottom left plot of group (a 'group' is an analysis of identical markers).

☒ All plots are contour plots with outliers or pseudocolor plots.

☒ A numerical value for number of cells or percentage (with statistics) is provided.

Methodology

Sample preparation

Adherent mouse breast cancer cell lines generated for this study were prepared for analytical flow cytometry as follows: Cells

Sample preparation

were harvested by trypsinization, PBS-washed, and resuspended in FACS buffer (PBS, 2% FCS, and 5 mM EDTA). In most experiments the detected fluoresences were present endogenously. For apoptosis assays cells were resuspendend in Annexin-V-binding buffer (0.1 M HEPES (pH 7.4), 1.4 M NaCl, and 25 mM CaCl2) at a concentration of 1 x 10^6 cells/ml, incubated with Annexin-V-FITC (BD, 556419) for 15 min at room temperature (RT) and subsequently with 7-AAD (eBioscience, 00699350) for 5 min at RT.
Cells from orthotopically transplanted tumors were isolated by mechanical and enzymatic dissociation. Subsequently, the cell suspension was filtered using 100 and 40 µm cell strainers and washed twice with PBS. The samples were measuerd without further processing as the cells endogenously express the flourescent proteins.

Instrument

LSR II flow cytometer (BD Bioscience)

Software

FACSDiva and FlowJo software (both BD Bioscience)

Cell population abundance

The study did not involve low abundant subpopulations of cells. Flow cytometry was used as an analytical tool for standard cell assays, i.e. competitive growth assays, label-retention assays, total lysosomal proteolysis, lysotracker staining, and Annexin-V binding/7-AAD uptake to measure apoptosis. There was no cell sorting.

Gating strategy

Cell debris and doublets were excluded using forward and side scatter. Further analysis depended on the experiment (see Supplementary Fig. 11 for all gating strategies). For competitive growth assays the ratio of Tomato+ (non-recombined) cells to Tomato+GFP+ and GFP+ (recombined) cells was assessed. For apoptosis assays the amount of Annexin-V-7-AAD- (living) cells relative to DMSO-treated control and normalized to Ctsd+/+ cells is given; staurosporine-treated cells served as positive control for Annexin-V binding and 7-AAD uptake. For label-retention assays eFluor™ 670 intensity in PI- cells was divided into negative, low, and high to distinguish between fast-, slow-, and non-proliferating (label-retaining) cells, respectively. Here, unstained and freshly stained cells were used as controls. For the assessment of acidic compartments in live cells, LysoTrackerTM Green intensity in PI- cells was divided into negative, low, medium, and high. Unstained and Torin-treated cells served as controls. For lysosomal activity assays the recovery of fluorescence from the self-quenched substrate was quantified by the median fluorescence intensity. Here, cells incubated with Cytochalasin D, Bafilomycin A1 or without any substrate served as controls.

☒ Tick this box to confirm that a figure exemplifying the gating strategy is provided in the Supplementary Information.
